# Geostationary satellite observations of extreme and transient methane emissions from oil and gas infrastructure

**DOI:** 10.1073/pnas.2310797120

**Published:** 2023-12-19

**Authors:** Marc Watine-Guiu, Daniel J. Varon, Itziar Irakulis-Loitxate, Nicholas Balasus, Daniel J. Jacob

**Affiliations:** ^a^School of Engineering and Applied Sciences, Harvard University, Cambridge, MA 02138; ^b^Department of Computer Science, ETH Zürich, Zürich 8092, Switzerland; ^c^Research Institute of Water and Environmental Engineering, Universitat Politècnica de València, Valencia 46022, Spain; ^d^International Methane Emissions Observatory, United Nations Environment Programme, Paris 75015, France

**Keywords:** methane, geostationary, satellites, remote sensing

## Abstract

We demonstrate geostationary satellite monitoring of large transient methane point sources with the US Geostationary Operational Environmental Satellites (GOES). GOES provides continuous 5- to 10-min coverage of the Americas at 1 to 2 km nadir pixel resolution in two shortwave infrared spectral bands from which large methane plumes can be retrieved. We track the full evolution of an extreme methane release from the El Encino—La Laguna natural gas pipeline in Durango, Mexico on 12 May 2019. The release lasted 3 h at a variable rate of 260 to 550 metric tons of methane per hour and totaled 1,130 to 1,380 metric tons. We report several other detections of transient point sources from oil/gas infrastructure, from which we infer a detection limit of 10 to 100 t h^−1^. Our results show that extreme releases of methane can last less than an hour, as from deliberate venting, and would thus be difficult to identify and quantify with low-Earth orbit satellites.

## Significance

Decreasing atmospheric methane emissions is an urgent priority to slow near-term climate change. Satellites have unique capabilities to pinpoint methane sources in support of climate action, but the current observing system is entirely in low-Earth orbit and thus has difficulty identifying and quantifying transient emissions. Here, we demonstrate continuous 5-min monitoring of large methane point sources with the U.S. Geostationary Operational Environmental Satellites (GOES). We apply this to quantify an extreme 3-h methane release from a natural gas pipeline in Durango, Mexico. We detect other large releases lasting less than 1 h from oil and gas infrastructure. Our results demonstrate the potential for continuous monitoring of large methane point sources from geostationary orbit.

Methane is a short-lived greenhouse gas responsible for about one-third of greenhouse radiative forcing since preindustrial times (1). Anthropogenic methane emissions include major contributions from agriculture, oil and gas production, coal mining, and waste (2). Mitigating methane emissions has become increasingly attractive as a strategy to slow near-term climate change (3), with momentum building in recent years behind the 2021 Global Methane Pledge signed by more than 100 countries, the 2022 US Inflation Reduction Act with measures to limit methane emissions from oil and gas production, and the 2023 Fit for 55 plan to slash European Union greenhouse gas emissions 55% from 1990 levels by 2030, among other efforts.

Satellite instruments sensitive to atmospheric methane have unique capabilities for monitoring emissions in support of climate action. They can quantify methane emissions from the global scale down to individual point sources by observing back-scattered sunlight in the shortwave infrared (SWIR; 4, 5). However, the current fleet is entirely in low-Earth orbit (LEO), with revisit rates for areas of interest of 1 d at best. All but one of the instruments (the Earth Surface Mineral Dust Source Investigation, EMIT; 6) are sun-synchronous, with overpasses between 9:30 and 13:00 local solar time. This means that the current observing system is largely incapable of monitoring the diurnal variability and intermittency of methane emissions, which can lead to substantial bias in emission estimates (7–9).

Here, we show how the US Geostationary Operational Environmental Satellites (GOES) with 1 to 2 km nadir pixel resolution in their SWIR bands can monitor very large methane point sources in the Americas with up to 5-min continuous coverage. We apply this capability to track the full evolution of an extreme methane release from the El Encino—La Laguna (EELL) natural gas pipeline in Durango, Mexico on 12 May 2019. We also detect additional releases indicating that these can be very brief, as from deliberate venting, and easily misinterpreted in terms of source location and magnitude by LEO instruments.

## Results

**Fig. 1.**
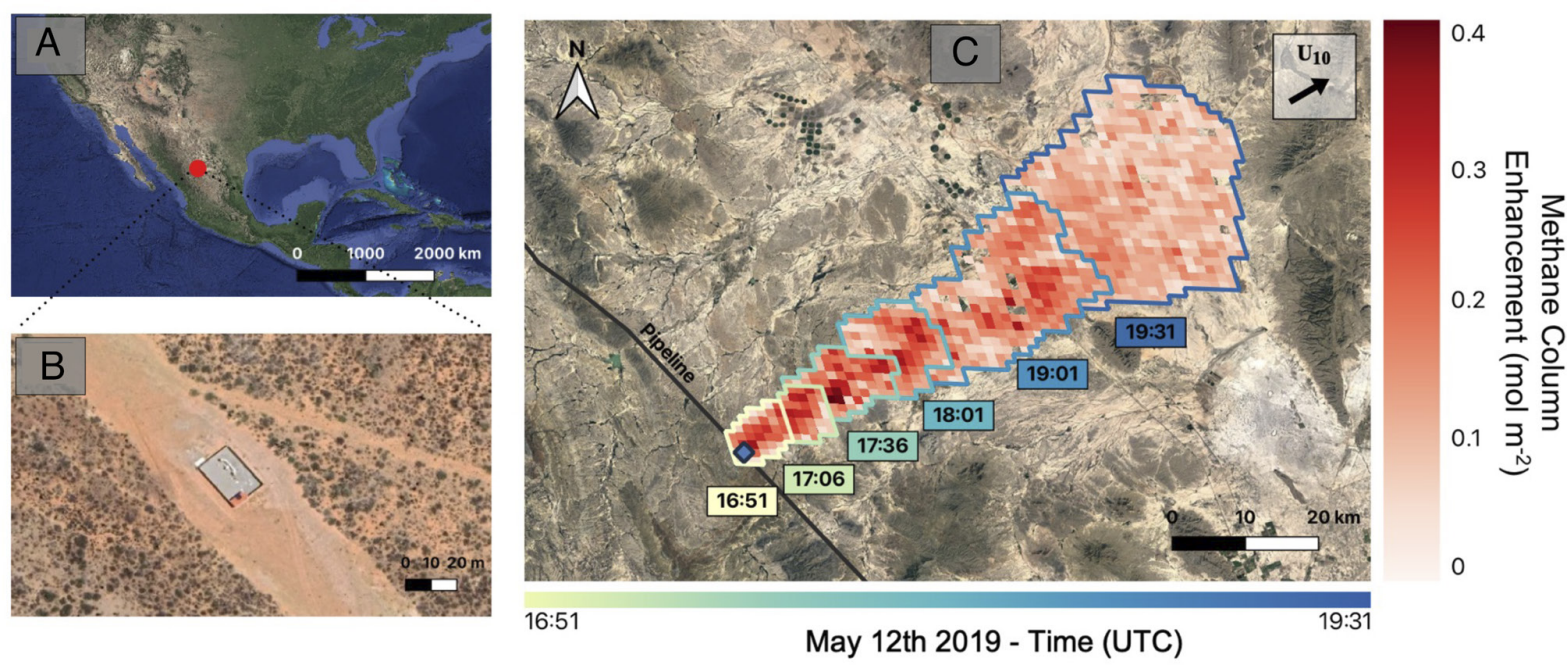
GOES satellite detection of a large methane release along the EELL natural gas pipeline in northern Mexico on 12 May 2019. (*A*) Location of the source in Durango, Mexico. (*B*) The pipeline block valve station responsible for the release (26.08580°N, 104.31682°W). (*C*) Temporal progression of the methane plume illustrated by 5 selected snapshots from 16:51 to 19:31 UTC on 12 May 2019. The snapshots are overlaid in reverse chronological order (earliest on top) and outlined according to acquisition time. The 10-m wind vector (***U***_10_) is from the GEOS-FP meteorological reanalysis product at 0.25° × 0.3125° resolution (14). The pipeline path (black line) is from the Global Energy Monitor Wiki (May 2023), and background imagery is from © (2023) Google Earth.

The EELL pipeline release was first observed by the TROPOspheric Monitoring Instrument on 12 May 2019 (TROPOMI; 10; *SI Appendix*, Fig. S1) and reported at 372 t h^−1^ by Lauvaux et al. (9). This is comparable in instantaneous magnitude to historic methane releases such as the 2022 Nord Stream pipeline rupture (11). The EELL pipeline is part of the Wahalajara pipeline system that supplies Mexico with natural gas from the US Permian Basin (12). It transports 1.5 Bcf per day of natural gas from Chihuahua to Durango. On 11 May 2019, Sentinel-2 (*SI Appendix*, Fig. S2) also detected large emissions near the pipeline and identified the source as a pipeline block valve station (26.08580°N, 104.31682°W; Fig. 1 and *SI Appendix*, Fig. S2). Block valve stations are used to isolate sections of natural gas pipelines during maintenance, emergency shutdowns, and release of excess pressure (13). We focus our analysis here on GOES-East imagery of the EELL pipeline in Durango, with a viewing zenith angle of 23° and resulting pixel resolutions of 1.3 km and 2.5 km in the SWIR bands 5 and 6 from which we perform our GOES methane retrievals (*Materials and Methods*).

Fig. 1 shows the EELL pipeline source location along with a composite of GOES methane plume retrievals on 12 May 2019. Fig. 1 *A* and *B* show the location of the scene in northern Mexico and high-resolution surface imagery of the block valve station responsible for the observed methane release. Fig. 1*C* illustrates the temporal evolution of the 12 May 2019 release with five hand-selected GOES snapshots of the resulting methane plume (Movies S1 and S2). The release lasted 3 h, after which the plume, extending more than 80 km downwind, detached from the source. The peak plume enhancement was 1,300 ppb during this period, 70% above atmospheric background levels. *SI Appendix*, Fig. S3 shows 48 GOES plume retrievals at 5-min intervals over the full course of the event.

**Fig. 2.**
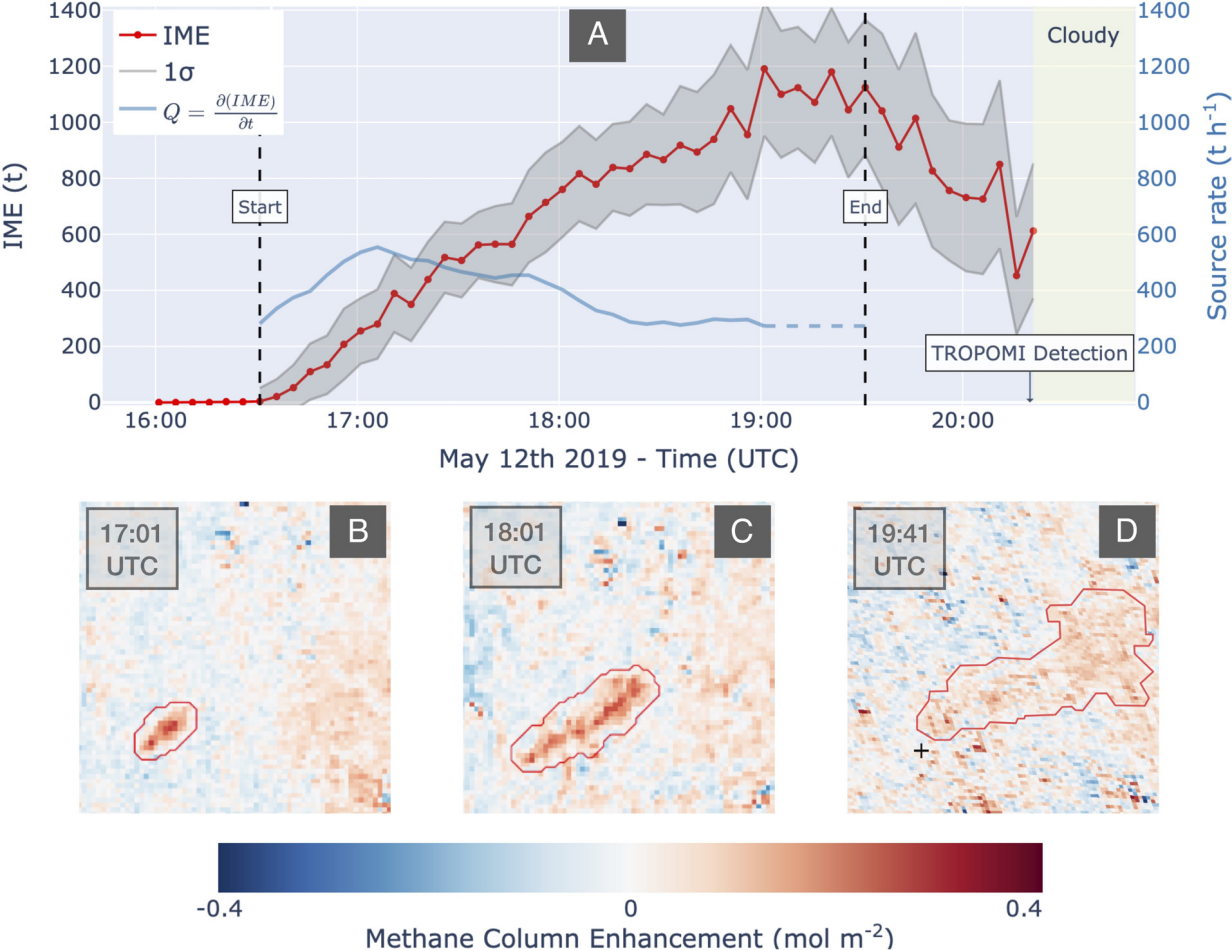
GOES methane plume retrievals and source rate quantification for the Durango EELL pipeline source of Fig. 1 on 12 May 2019. (*A*) 5-min evolution of the IME from which the source rate is inferred. The red line shows the evolution of the integrated methane enhancement (IME) from the sequence of plume images and the gray envelope gives the associated 1-sigma uncertainty. The blue line estimates the time-dependent source rate Q (right axis) as the trend of a 5-point moving average of the IME from 16:30 to 19:00 UTC, with the dashed segment indicating extrapolation. The dashed vertical lines mark the start and end of the release. (*B*–*D*) Sample methane retrieval fields and plume masks (red contour lines) showing the temporal evolution of the imaged plume from 17:01 to 19:41 UTC.

Fig. 2 shows our quantification of the release mass and source rate. No plume was observed before 16:30 UTC. The release began at 16:30 UTC (11:30 local time) and ended at 19:30 UTC (14:30 local time), when the plume was observed to detach from the source (Fig. 2*D*). Clouds prevented viewing after 20:20 UTC. Sample multi-band–multi-pass (MBMP) methane retrieval fields are shown in Fig. 2 *B*–*D* along with the resulting plume masks (*Materials and Methods*). The methane plume is clearly detectable above a noise floor with a SD of 0.06 mol m^−2^ (~9% precision), and the plume masks capture its spatiotemporal evolution. Fig. 2*A* shows the growth in the integrated methane enhancement [IME (kg); *Materials and Methods*] over time. The IME increases rapidly in the first 2.5 h of the release with an average source rate of 460 ± 90 t h^−1^ (slope of regression line), comparable to the 372 t h^−1^ reported by Lauvaux et al. (9) from a single TROPOMI pass roughly 1 h after the release ended. It stabilizes in the third hour around 19:00, as distant plume enhancements begin to diffuse below the mask detection limit. It then declines around 19:30 UTC following the end of the release. The blue line shows our estimate of the time-dependent source rate as the trend of a 5-point moving average of the IME. Emissions fluctuated between 260 and 550 t h^−1^, with a gradual decline over the 2.5-h fitting period. Extrapolating these source rate estimates over the full release (dashed blue line) yields similar estimates of total methane emissions, with 1,380 t from the 460 t h^−1^ source rate and 1,130 t from the time-dependent source rate (area under curve). Assuming 80% methane content for Permian natural gas (15), this is enough to power 3,600 to 4,400 Mexican urban households (16) for a year.

We selected the 12 May 2019 release as a case study because it produced the strongest methane plume in the Lauvaux et al. (9) TROPOMI record. However, it was not an isolated incident, as evidenced by the previous day’s Sentinel-2 detection (*SI Appendix*, Fig. S2). In fact, TROPOMI and Sentinel-2 detected large methane plumes from three different block valve stations along the EELL pipeline on 11 different days between 7 April 2019 and 24 May 2019 (*SI Appendix*, Figs. S4–S6). Five of the releases are from the block valve station shown in Fig. 2, and the others occurred at stations within 65 km of that source on 6 other days (7 April 2019; 16 and 21 to 24 May 2019; *SI Appendix*, Figs. S4–S6). GOES detects emissions for all events, but cloud cover prevents quantification over the full durations of the releases except on 12 May 2019. We estimate source rates of ~140 to 340 t h^−1^ from cloud-free GOES coverage on the other days (*SI Appendix*, Fig. S6), demonstrating the unique capability of geostationary satellite instruments to observe during cloud-free periods of otherwise cloudy days.

## Discussion

Our work illustrates the potential of geostationary satellites to provide continuous monitoring of large methane point sources. We showed how 5-min GOES imagery enabled quantification of the duration, source variability, and total emission of a natural gas pipeline methane release previously detected by Lauvaux et al. (9) as a single snapshot from TROPOMI observations. TROPOMI observed the plume roughly 50 min after the end of the release with an aliased origin more than 10 km downwind of the actual source location and just before clouds set in. The continuous imaging with GOES illustrates the improved ability of geostationary observations to localize and quantify intermittent sources. Lacking better information, Lauvaux et al. (9) assumed a default 24-h duration for their instantaneous TROPOMI methane plumes observed on consecutive days to estimate total annual emissions. We show here that such events can occur over much shorter periods of time and with variable source rate, even when observed in the same location on consecutive days. LEO satellites cannot effectively quantify the total emissions from transient point sources.

The 2019 EELL pipeline releases were extreme and their detection with GOES was facilitated by prior knowledge from TROPOMI and Sentinel-2. Other large plumes can be detected in the GOES record by cross-referencing with TROPOMI. For example, GOES traces back two large methane plumes observed by TROPOMI over Illinois on 15 June 2020 to simultaneous natural gas pipeline releases (440 to 550 t h^−1^ for 1 h) in Indiana, 5 h earlier and 60 km upwind (*SI Appendix*, Fig. S7 and Movie S3). Independently of other satellite observations, we performed a visual survey with GOES over January and July 2023 of a 100 × 100 km^2^ subregion of the Permian basin (30.950°N to 31.879°N, 103.051°W to 104.082°W) containing extensive oil and gas infrastructure. By visual inspection of the scans from these periods, we identified one methane release from a cluster of production facilities (31.552°N, 103.843°W on 26 July 2023), which lasted less than 30 min at a rate of 170 t h^−1^ (*SI Appendix*, Fig. S8 and Movie S4). Such a brief event is unlikely to be recorded by LEO instruments, and this one had not previously been detected. The GOES SWIR pixel resolution of 2 km is too coarse to identify the emitting facility within the cluster. Signal to noise for the detection is relatively high (>4.5), with background retrieval precision of ~0.036 mol m^−2^ compared to a peak plume concentration of 0.167 mol m^−2^. We therefore estimate a detection limit in the range 10 to 100 t h^−1^ for our GOES methane retrievals. This detection limit could be improved with a better algorithm for selecting reference background scenes (*Materials and Methods*).

The retrieval techniques we present here for GOES can be readily applied to other geostationary satellite systems with similar SWIR bands and revisit rates, including the Japanese Himawari constellation over Asia and Oceania (17) and the European Meteosat Third Generation constellation over Europe, Africa, and the Middle East (18). More work is needed to characterize the methane sensitivity of these instruments under different observing conditions, including variable surface types and sun-satellite geometries, and to what extent the methane retrievals can be improved to detect smaller sources. More systematic scanning of the GOES record for large methane plumes, both independently and exploiting information from other satellite instruments, is another avenue for future research.

**Fig. 3.**
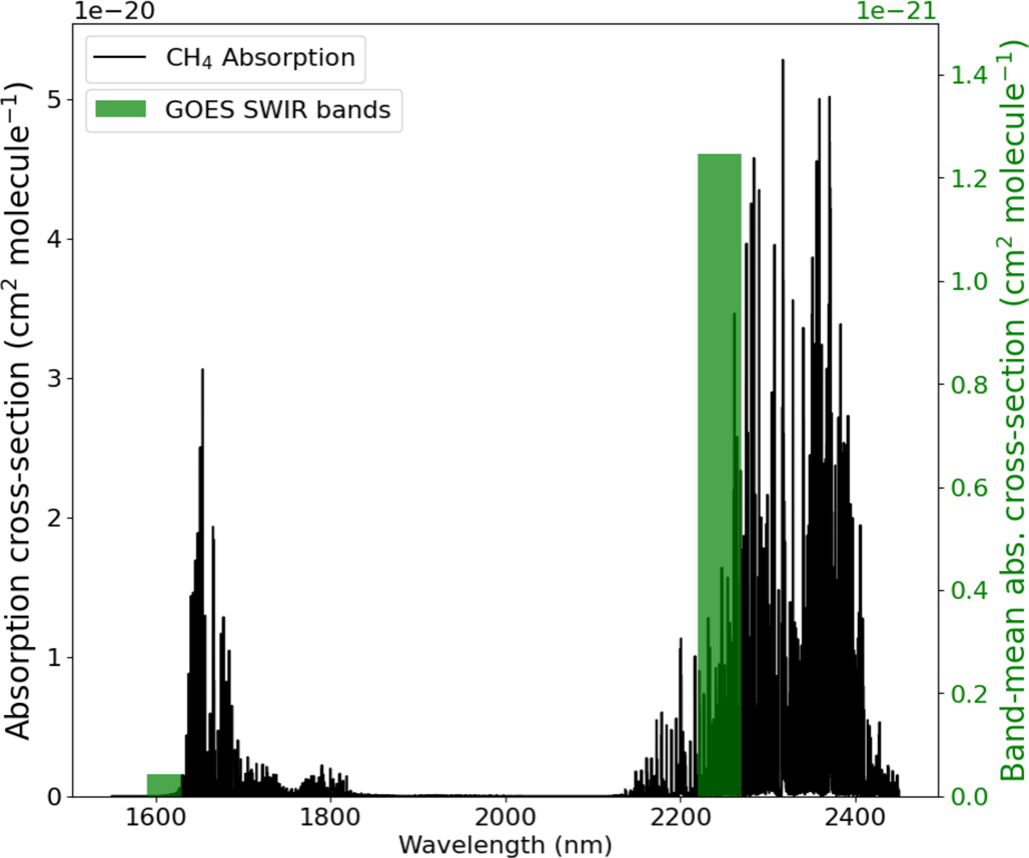
Methane absorption cross-sections and GOES bands (5 and 6) in the SWIR spectral range. The absorption cross-sections (left axis) are from HITRAN2016 line spectra convolved with a Voigt profile (20). The mean absorption cross-section in each GOES band is plotted on the right axis.

Our results highlight the importance of developing dedicated geostationary satellite instruments to monitor greenhouse gasses. The GeoCarb instrument (19) planned for launch to geostationary orbit in 2025 was recently canceled. It would have provided daily observation of methane concentrations at 10 km spatial resolution over the Americas with highly resolved SWIR spectra. Future development of geostationary instruments for methane monitoring should balance the needs for rapid revisits, high spatial resolution, and fine spectral sampling. Subhourly revisits are needed to detect brief releases and accurately quantify their total duration and mass, while source detection limits improve with spatial and spectral resolution. More work is needed to determine the optimal SWIR band configuration for detecting methane in future multispectral instruments. As shown in Fig. 3, the position of GOES SWIR band 6 could be adjusted for much-improved methane detection. Until a successor mission is launched with better methane sensitivity, GOES can be of great value for near-real-time detection of extreme methane point sources.

## Materials and Methods

### GOES Satellite Observations.

GOES is a geostationary satellite constellation operated by the US National Oceanic and Atmospheric Administration to monitor the land and atmosphere across the Americas. It comprises two satellites, GOES-East at 75.2°W and GOES-West at 137.2°W, which together provide full-disk (hemispheric) scans every 10 min and scans of the continental United States and parts of Canada and Mexico every 5 min. The GOES Advanced Baseline Imager (ABI) delivers multispectral imagery in 16 spectral bands from the visible to thermal infrared, with nadir pixel resolution of 0.5 to 2 km depending on the band (21). Here, we retrieve methane column enhancements (mol m^−2^) from GOES ABI Level-1B (L1B) top-of-atmosphere reflectances in the SWIR bands 5 (~1,590 to 1,630 nm) and 6 (~2,220 to 2,270 nm), which have respective nadir pixel resolutions of 1 km and 2 km. The L1B GOES data are open-access and were obtained from https://noaa-goes16.s3.amazonaws.com/index.html#ABI-L1b-RadC/.

Fig. 3 shows methane absorption cross-sections in the 1,550 to 2,450 nm SWIR spectral range, based on absorption line spectra from the high-resolution transmission (HITRAN2016; 20, 22) molecular absorption database. Also shown are the GOES SWIR bands (bands 5 and 6), including position, width, and mean absorption cross-section. Band positions are comparable to those of the Sentinel-2 (20 m pixels) and Sentinel-3 (500 m pixels) multispectral satellite instruments with demonstrated methane sensitivity to large point sources (23–25).

### GOES Methane Retrieval.

We apply the MBMP methane retrieval of Varon et al. (23) to infer methane column enhancements (mol m^−2^) from normalized reflectance differences between GOES bands 5 and 6 on different 5-min scans of the scene of interest. In the case of the EELL pipeline scene, GOES performed 84 scans of the study area between 14:00 and 21:00 UTC (9:00 to 16:00 local time) on 12 May 2019. For a scan of interest (target scan), the MBMP method requires a plume-free reference scan under similar observing conditions with which to remove surface-related artifacts from the retrieval. For each scan starting at 14:00 UTC, we construct a reference scan from the average of the 7 scans that occurred within 15 min of the target scan time on another day when no significant methane enhancement was observed. We use the GOES L2 cloud mask product to select the most recent such day that was cloud-free. Averaging multiple scans reduces random noise in the retrieval.

The retrieval compares the target-scan reflectances $R_{5}$ and $R_{6}$ with the reference-scan reflectances $R_{5}^{\prime }$ and $R_{6}^{\prime }$ . The first step is to compute the fractional difference in reflectance between bands for the target and reference scans:

[1] $$\Delta R=\frac{cR_{6}-R_{5}}{R_{5}},$$

[2] $$\Delta R^{\prime }=\frac{c^{\prime }{R^{\prime }}_{6}-{R^{\prime }}_{5}}{{R^{\prime }}_{5}}$$

where *c* and *c’* are scaling factors to remove scene-wide brightness differences between bands 5 and 6. They are calculated by linear regression fitting of all *R*_6_ values against all *R*_5_ values across the scene (23). To compute methane column enhancements ΔΩ (mol m^−2^), we compare ΔR and $\Delta R^{'}$ to a fractional absorption model:

[3] $$m\left(\Delta \Omega \right)=\frac{T_{6}\left(\Omega +\Delta \Omega \right)}{T_{6}\left(\Omega \right)}-\frac{T_{5}\left(\Omega +\Delta \Omega \right)}{T_{5}\left(\Omega \right)},$$

where T   is the modeled top-of-atmosphere radiance, and the second term on the right-hand side accounts for some absorption by methane in band 5 (Fig. 3). We use the 100-layer, clear-sky radiative transfer model of Varon et al. (23) to simulate $T_{5}$   and $T_{6}$   in the GOES SWIR bands 5 (1,590 to 1,630) and 6 (2,220 to 2,300) at 0.02 nm resolution, accounting for variable viewing/solar zenith angles and integrating over each band’s spectral window. The model considers vertical profiles of methane, water vapor, and CO_2_, from the US Standard Atmosphere (26). Absorption line spectra are from HITRAN2016. We use a Newton method to solve for ΔΩ and for $\Delta \Omega^{\prime }$ by minimizing $\left|\Delta R-m(\Delta \Omega )\right|$ and $\left|\left.\Delta R^{\prime }-m(\Delta \Omega^{\prime })\right|\right.$ and subtract the results to compute the MBMP methane enhancement:

[4] $$\Delta \Omega_{\text{MBMP}}=\Delta \Omega -\Delta \Omega^{\prime }.$$

This subtraction removes systematic surface-related errors in the retrieval by deleting artifacts present in both retrieval fields.

We use the GOES Clear Sky Mask (ACM) Level 2 product to mask out clouds when computing ΔR and $\Delta R^{'}$ . Small clouds sometimes go undetected and introduce error in the retrieval (e.g., *Bottom* row of *SI Appendix*, Fig. S3). Ground shadows from the clouds are another source of retrieval artifacts. Other artifacts arise directly from surface features, such as the seemingly methane-enhanced areas in the southeastern quadrant of the panels in *SI Appendix*, Fig. S3. We can discard these because they do not look like plumes and are not transported with the wind. Future work could attempt to filter these out using metrics like the structural similarity index (SSIMl; 27) between retrieval and reference images, as proposed by Pandey et al. (25) for Sentinel-3. To avoid false positives from nonmethane plumes, such as smoke from a flare, we separately compute a pseudo-retrieval replacing the methane-sensitive band 6 with a visible band; if a plume appears in the pseudo-retrieval, we reject it as an artifact.

### Source Rate Retrieval.

Quantifying the source rate of a methane point source from satellite plume imagery first requires constructing a binary plume mask to separate methane enhancements from background noise. We mask our GOES methane plumes by hysteresis thresholding with low and high threshold (28). This incorporates pixels above the low threshold (50th percentile) when they are connected to pixels above the high threshold (95th percentile), including in the previous snapshot. We find that this dual-threshold approach smooths the evolution of the mask from scan to scan. We further apply a 3x3 median filter and manual postprocessing as needed to smooth the mask edges.

Standard methods for inferring point source rates from the observation of instantaneous plumes, including the cross-sectional flux (CSF) and IME methods, generally assume a constant source over the lifetime of the plume within the mask (5, 29). This is not the case in our GOES observations where the sources are transient and the plumes grow with time before dissipating. We use here a different method by tracking the evolution of the IME within the mask for individual scans. The IME (kg) for a given scan is computed as (29, 30)

[5] $$\text{IME}=M_{{\text{CH}}_{4}}\sum_{j=1}^{N}\Delta \Omega_{j}A_{j},$$

where $M_{{\text{CH}}_{4}}=0.01604$   (kg mol^−1^) is the molar mass of methane, $\Delta \Omega_{j}$   (mol m^−2^) is the methane column enhancement in the *j*th plume pixel, $A_{j}$   (m^2^) is the corresponding pixel area, and *N* is the total number of masked pixels. We estimate the source rate from the growth of IME over time in two different ways: 1) as the slope of an ordinary least-squares regression line, which estimates the period-mean source rate, and 2) from the trend of the IME time series, which captures the time-dependent source rate. These approaches have the advantage of being independent of the local wind speed, which is typically the primary uncertainty in source rates retrieved from LEO satellite observations (29, 31). A disadvantage is that they do not account for loss by outflow through the mask edges, which would bias the source rate low. This bias should be small for large sources during the plume’s initial growth phase, when the plume is highly concentrated and easily distinguishable from the background (Movies S1 and S2). They are also not applicable to steady-state sources, which are better interpreted using the wind-based IME and CSF methods. To estimate uncertainty in the source rate, we perform the masking and quantification procedure using reference scans from 6 different dates when no significant methane enhancement was observed over the location (13, 17, 18, 19, 20, and 21 May 2019 for the EELL case) and report the mean and SD of results across the 6 estimates.

We estimate the beginning and end of the release directly from the satellite data by monitoring the near-field IME within the 3x3 pixel neighborhood of the source. We define the source as active when the near-field IME exceeds 10 t, until the plume detaches from the source. Observing the detachment of the plume is important because the end of the release cannot be inferred solely from a decline in IME, which would also result from a variable emission that falls below the rate of outflow from the plume mask. We compute the source rate regression line from the beginning of the event until the observed IME stabilizes, indicating an approach to steady state when outflow from the plume mask balances the source rate. We diagnose this stabilization by visual inspection of the IME trend with time and extrapolate the variable source rate (dashed blue line in Fig. 2*A*) from that time (~19:00) to the end of the release.

## Supplementary Material

Appendix 01 (PDF)

Movie S1. 5-minute sequence of GOES (masked) methane plume retrievals for the 12 May 2019 EELL pipeline release. Background imagery is from © (2023) Google Earth.

Movie S2. 5-minute sequence of GOES (unmasked) methane plume retrievals for the 12 May 2019 EELL pipeline release.

Movie S3. 5-minute sequence of GOES methane plume retrievals for the 15 June 2020 simultaneous pipeline releases shown in Fig. S7.

Movie S4. 5-minute sequence of GOES methane plume retrievals for the 26 July 2023 Permian release shown in Fig. S8.

## Data Availability

GOES methane retrieval fields for all reported plumes have been deposited in the Harvard Dataverse (https://doi.org/10.7910/DVN/EQWHCG) (32).
