# Supplementary material for: Geostationary satellite observations of extreme and transient methane emissions from oil and gas infrastructure
Source: Proc Natl Acad Sci U S A. 2023 Dec 19;120(52):e2310797120. doi: 10.1073/pnas.2310797120 (PMC10756283; doi:10.1073/pnas.2310797120)
Supplement: Supplementary file 1 — Appendix 01 (PDF) [file pnas.2310797120.sapp.pdf]

PNAS

Supporting Information for

Geostationary satellite observations of extreme and transient methane emissions from oil and gas infrastructure

Marc Watine-Guiu[1,2*||†], Daniel J. Varon[1*||], Itziar Irakulis-Loitxate[3,4], Nicholas Balasus[1], Daniel J. Jacob[1]

[1] Harvard University, Cambridge, 02138, USA.
[2] ETH Zürich, 8092 Zürich, Switzerland.
[3] Universitat Politècnica de València, Valencia, Spain.
[4] International Methane Emissions Observatory, United Nations Environment Programme, Paris, France.

† Now at the United Nations Environment Programme International Methane Emissions Observatory (IMEO)

***Corresponding authors:** Marc Watine-Guiu and Daniel J. Varon

Email: marc.watine@un.org, danielvaron@g.harvard.edu

Author Contributions: || M.W.G. and D.J.V. contributed equally to this work.

Competing Interest Statement: The authors declare that they have no competing interests.

Classification: Physical Sciences.

Keywords: Methane, Geostationary, Satellites, Remote Sensing.

This PDF file includes:

Supporting figures S1 to S8
Captions for Supporting movies S1 to S5
References for Supporting Information

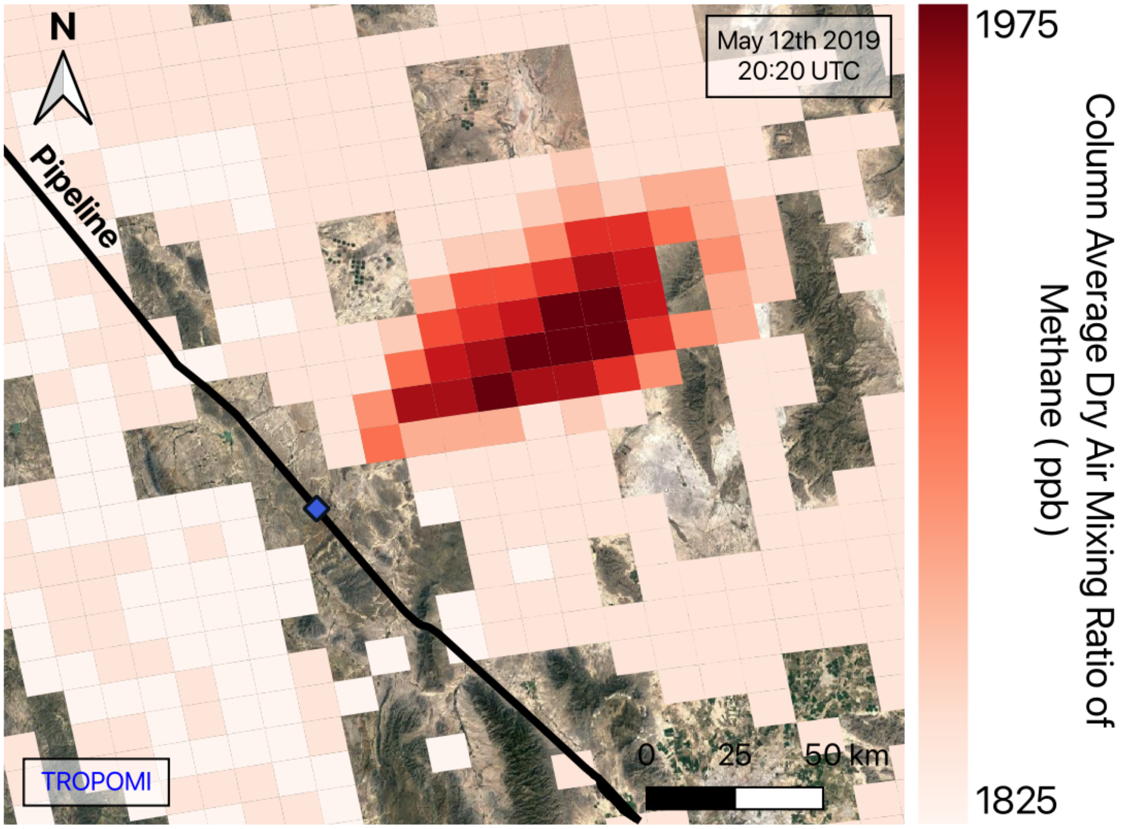

Figure S1. Methane plume from the EELL pipeline observed by TROPOMI (v02.04.00 operational data product) on 12 May 2019 at 20:20 UTC (15:20 local time), overlaid on surface imagery from © (2023) Google Earth.

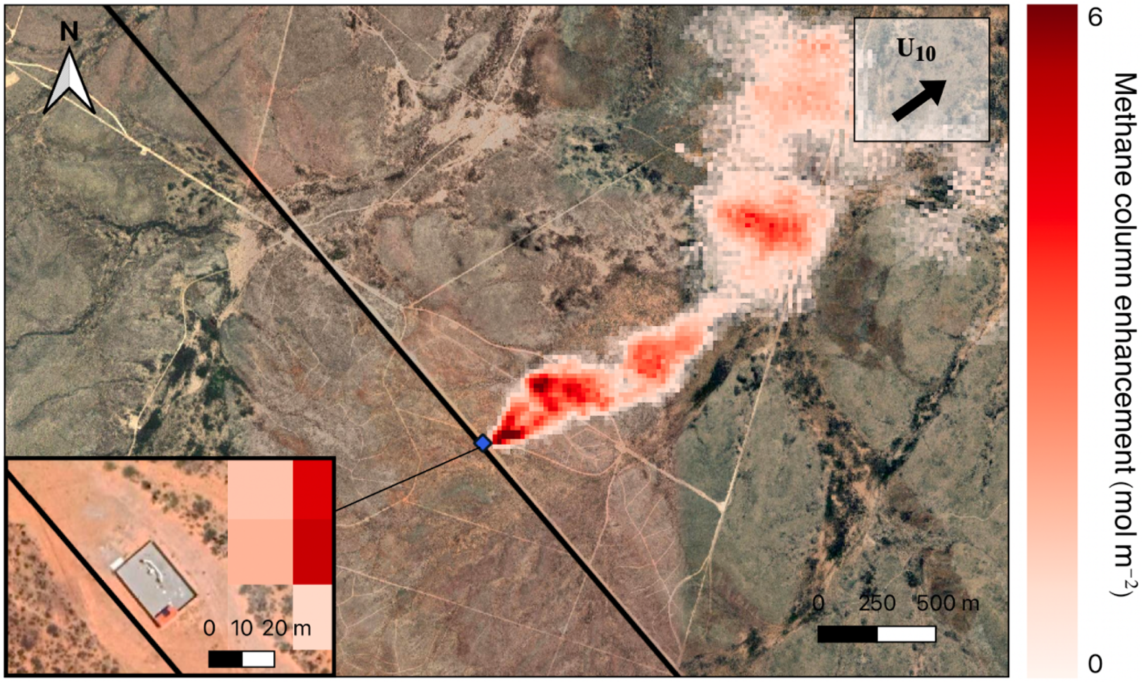

Figure S2. Sentinel-2 detection of large methane emissions from the EELL block valve station at (26.08580°N, 104.31682°W) on 11 May 2019 (12:46 local time). The methane retrieval was performed using the multi-band–multi-pass (MBMP) method (Varon et al., 2021; Irakulis-Loitxate et al., 2022). The emission was quantified at 551 ± 229 t h^{-1} by the IME method (Varon et al., 2018). The black line represents the EELL pipeline. Background imagery is from © (2023) Google Earth.

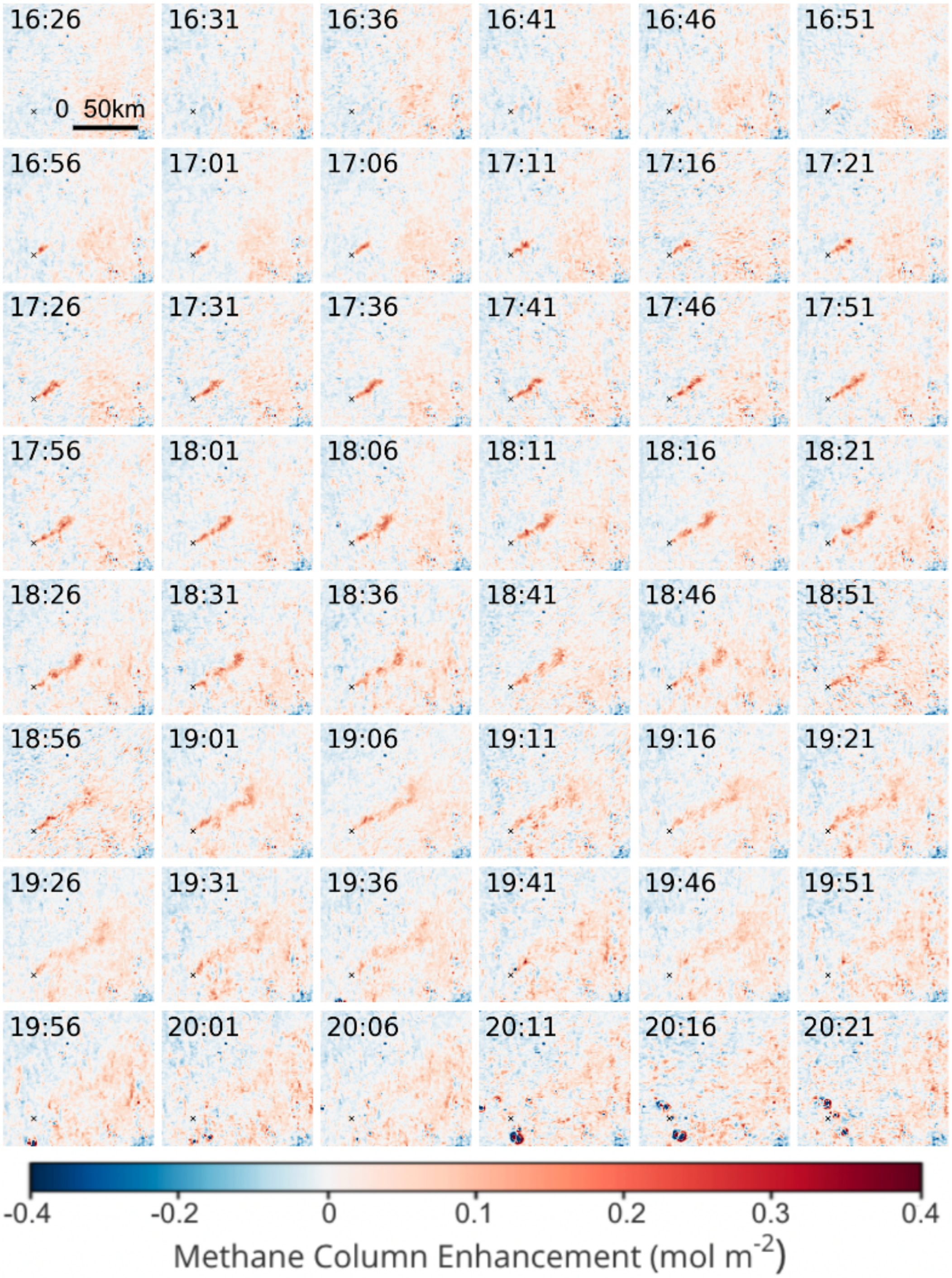

Figure S3. 48 snapshots of the EELL methane plume over the course of the 12 May 2019 release, from 16:26 to 20:21 UTC. The black x marks the source location. We estimate the release began at 16:30 UTC and ended at 19:30 UTC, after which point the plume detached from the source.

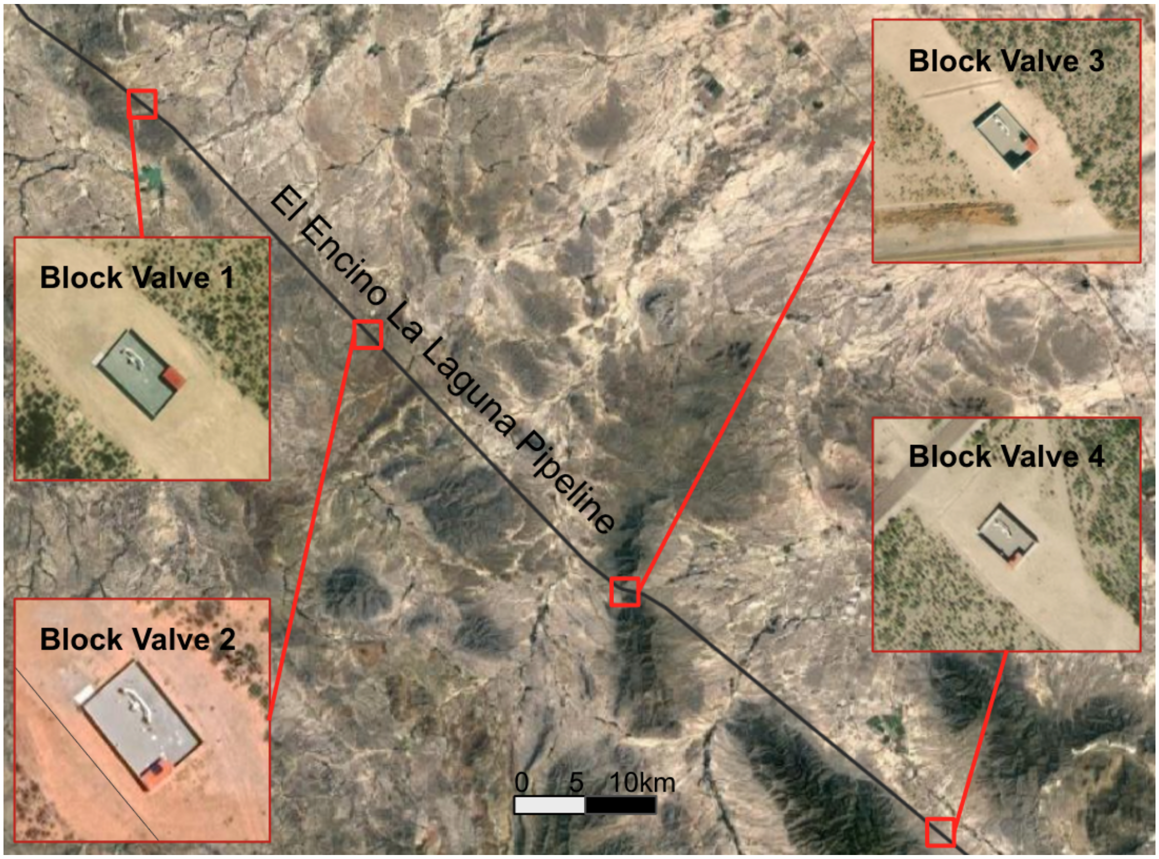

Figure S4. Four block valve stations analyzed with GOES on dates with TROPOMI or Sentinel-2 detections. They are spaced by roughly 30 km and located, from north to south, at (26.297686°N, 104.530079°W), (26.085840°N, 104.316830°W), (25.873025°N, 104.105993°W), and (25.681875°N, 103.855128°W). Block valve station 2 was the source of the large release detected by GOES on 12 May 2019. Background imagery is from © (2023) Google Earth.

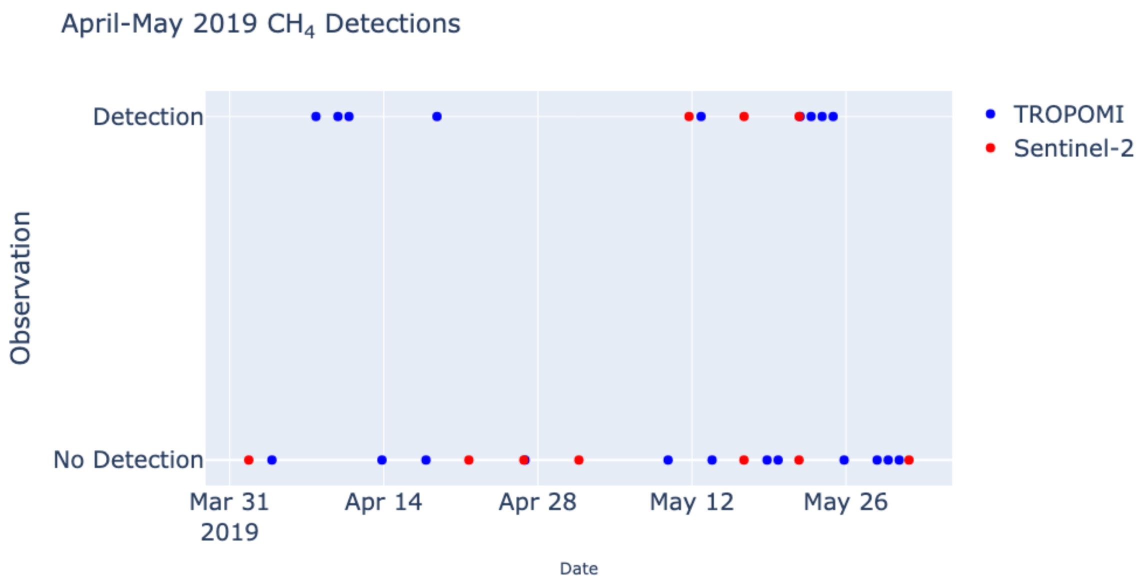

Figure S5. Temporal analysis of TROPOMI and Sentinel-2 detections/non-detections of EELL pipeline sources on cloud-free passes during April-May 2019. TROPOMI and Sentinel-2 detected large methane plumes on a combined 12 of 31 cloud-free passes. TROPOMI detections are based on the v02.04.00 operational data product. Sentinel-2 detections are based on the multi-band–multi-pass (MBMP) retrieval method (Varon et al., 2021; Irakulis-Loitxate et al., 2022).

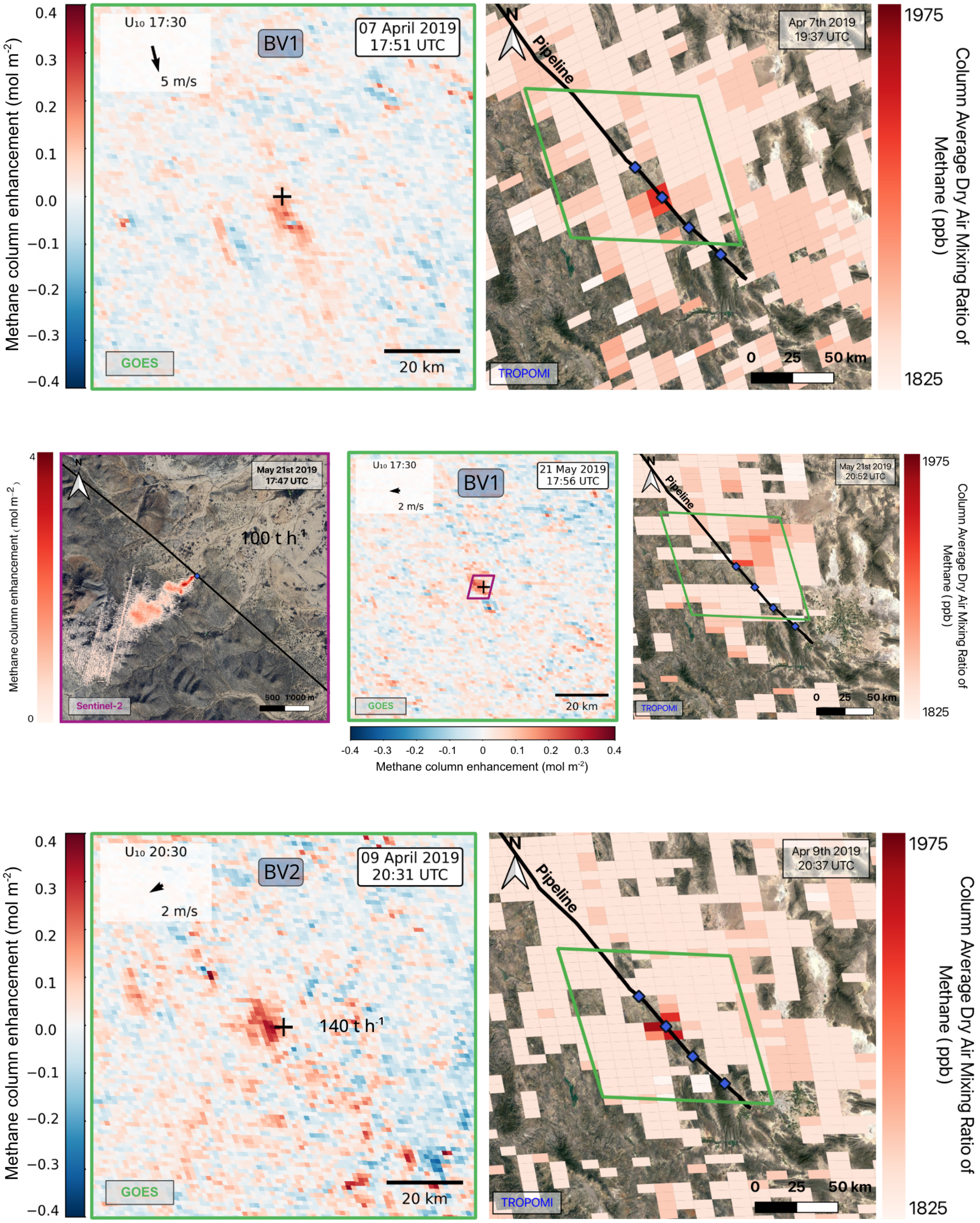

Methane column enhancement (mol m⁻²)
0.4
0.3
0.2
0.1
0.0
−0.1
−0.2
−0.3
−0.4
U₁₀ 17:30
5 m/s
BV1
07 April 2019
17:51 UTC
GOES
20 km
N
Pipeline
Apr 7th 2019
19:37 UTC
TROPOMI
0 25 50 km
1975
1825
Column Average Dry Air Mixing Ratio of Methane (ppb)
Methane column enhancement (mol m⁻²)
4
0
May 21st 2019
17:47 UTC
100 t h⁻¹
Sentinel-2
500 1'000 m
U₁₀ 17:30
2 m/s
BV1
21 May 2019
17:56 UTC
GOES
20 km
May 21st 2019
20:52 UTC
Pipeline
TROPOMI
0 25 50 km
1975
1825
Column Average Dry Air Mixing Ratio of Methane (ppb)
-0.4 -0.3 -0.2 -0.1 0 0.1 0.2 0.3 0.4
Methane column enhancement (mol m⁻²)
Methane column enhancement (mol m⁻²)
U₁₀ 20:30
2 m/s
BV2
09 April 2019
20:31 UTC
140 t h⁻¹
GOES
20 km
N
Pipeline
Apr 9th 2019
20:37 UTC
TROPOMI
0 25 50 km
1975
1825
Column Average Dry Air Mixing Ratio of Methane (ppb)

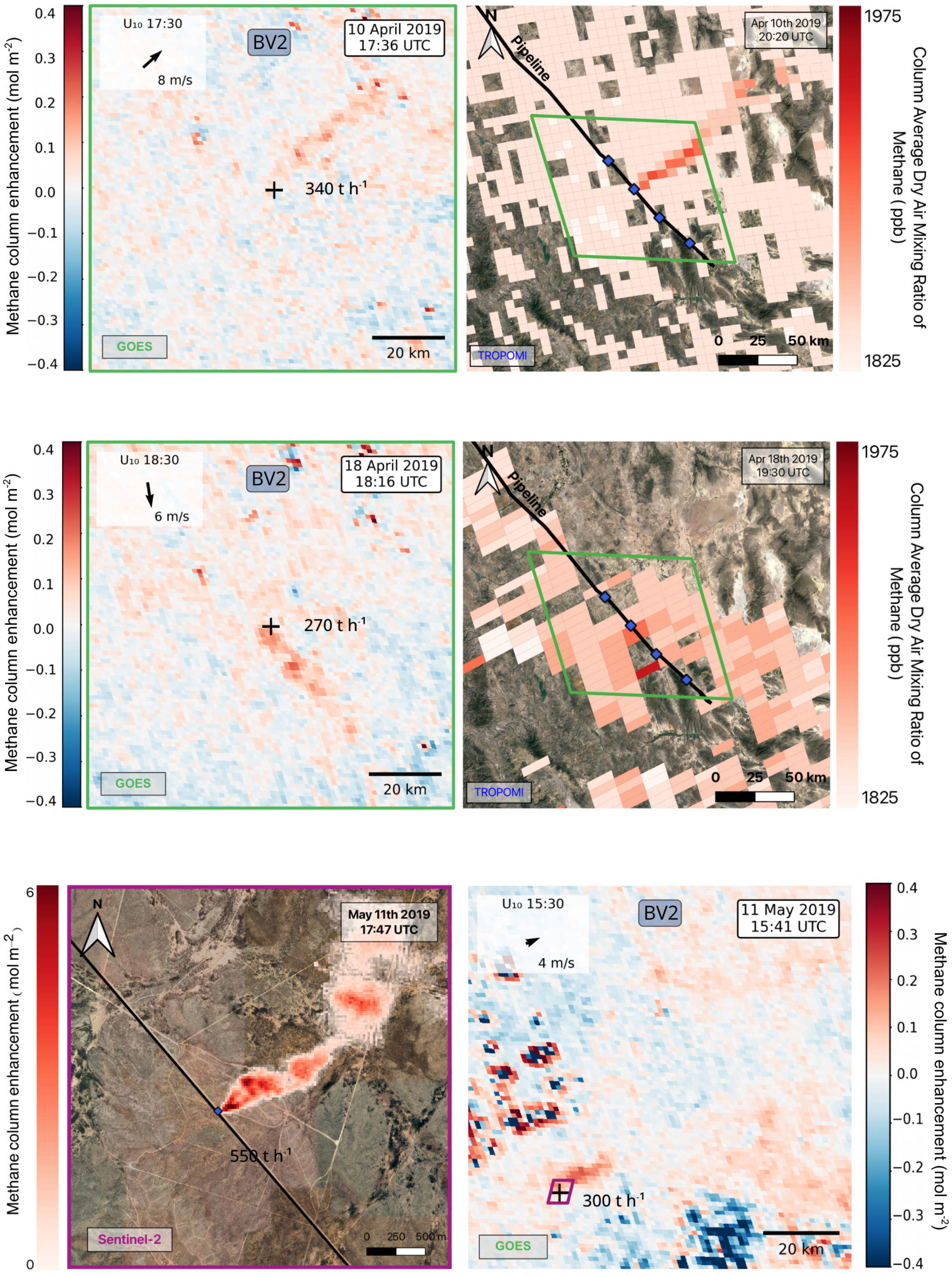

Methane column enhancement (mol m^{-2})
U10 17:30
8 m/s
BV2
10 April 2019 17:36 UTC
340 t h^{-1}
GOES
20 km
N
Pipeline
Apr 10th 2019 20:20 UTC
0 25 50 km
TROPOMI
Column Average Dry Air Mixing Ratio of Methane (ppb)
1975
1825
U10 18:30
6 m/s
18 April 2019 18:16 UTC
270 t h^{-1}
Apr 18th 2019 19:30 UTC
May 11th 2019 17:47 UTC
550 t h^{-1}
Sentinel-2
0 250 500 m
U10 15:30
4 m/s
11 May 2019 15:41 UTC
300 t h^{-1}

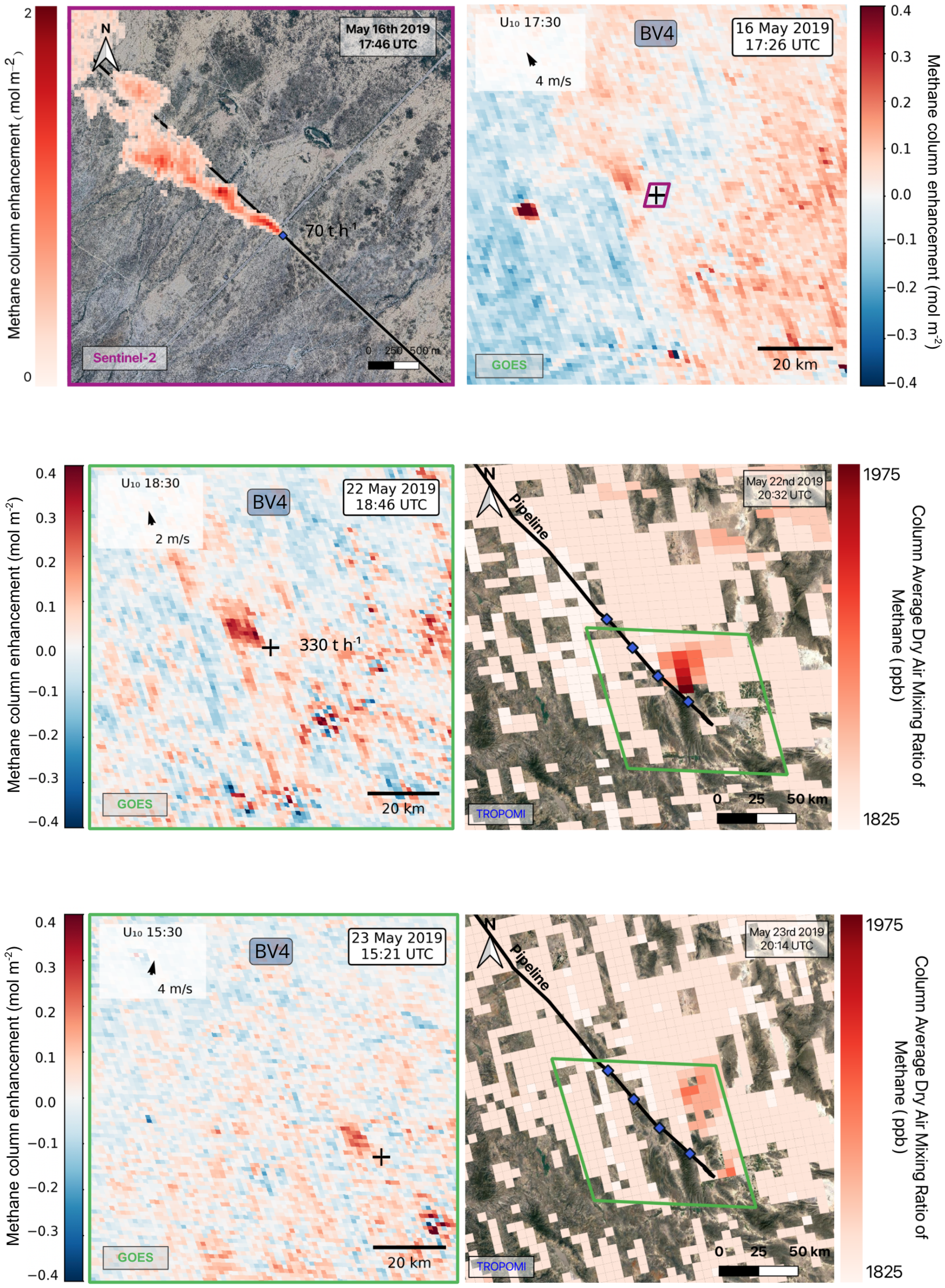

Methane column enhancement (mol m-2)
2
0
N
May 16th 2019
17:46 UTC
70 t h-1
Sentinel-2
0 250 500 m
U10 17:30
4 m/s
BV4
16 May 2019
17:26 UTC
20 km
GOES
0.4
0.3
0.2
0.1
0.0
−0.1
−0.2
−0.3
−0.4
Methane column enhancement (mol m-2)
U10 18:30
2 m/s
BV4
22 May 2019
18:46 UTC
330 t h-1
20 km
GOES
N
Pipeline
May 22nd 2019
20:32 UTC
0 25 50 km
TROPOMI
1975
1825
Column Average Dry Air Mixing Ratio of Methane (ppb)
U10 15:30
4 m/s
BV4
23 May 2019
15:21 UTC
20 km
GOES
N
Pipeline
May 23rd 2019
20:14 UTC
0 25 50 km
TROPOMI
1975
1825
Column Average Dry Air Mixing Ratio of Methane (ppb)

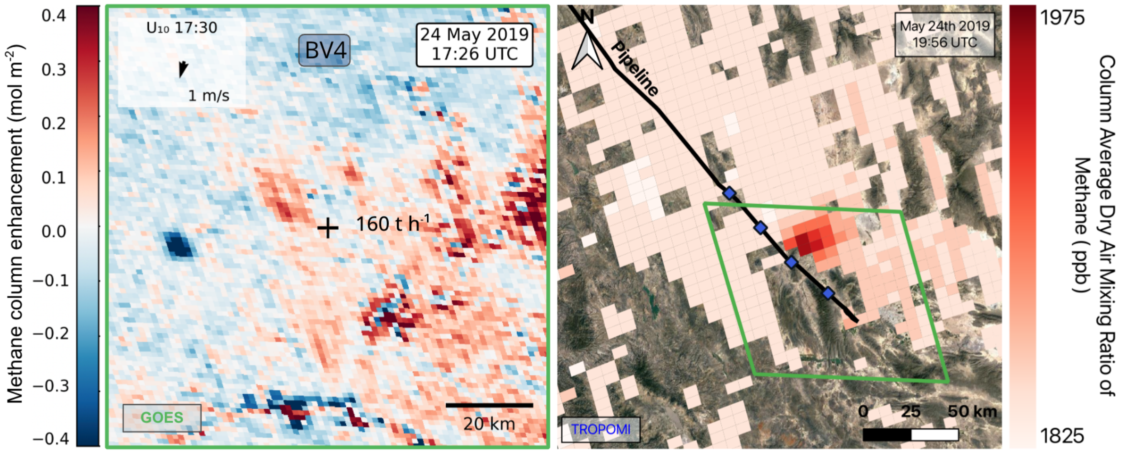

Figure S6. GOES, Sentinel-2, and TROPOMI methane plume detections from block valve stations 1 (BV1), 2 (BV2) and 4 (BV4) in April and May 2019 (see Fig. S4, S5), in addition to the 12 May 2019 detection discussed in the main text. The block valve stations are marked by black plus symbols in the GOES imagery and blue diamonds in the TROPOMI and Sentinel-2 imagery. No plumes were observed from block valve station 3 (Fig. S4). TROPOMI data are from the v02.04.00 operational data product. Sentinel-2 detections are based on the multi-band–multi-pass (MBMP) retrieval method (Varon et al., 2021; Irakulis-Loitxate et al., 2022). Source rates (inset) are reported from IME linear regressions during cloud-free periods where possible for GOES retrievals and using the wind-based IME method (Varon et al., 2018) for Sentinel-2 retrievals. GOES imaging domains are shown as green inset boxes in TROPOMI scenes; Sentinel-2 imaging domains are shown as pink inset boxes in GOES domains. Wind insets show the 10-m wind from GEOS-FP. Background imagery is from © (2023) Google Earth.

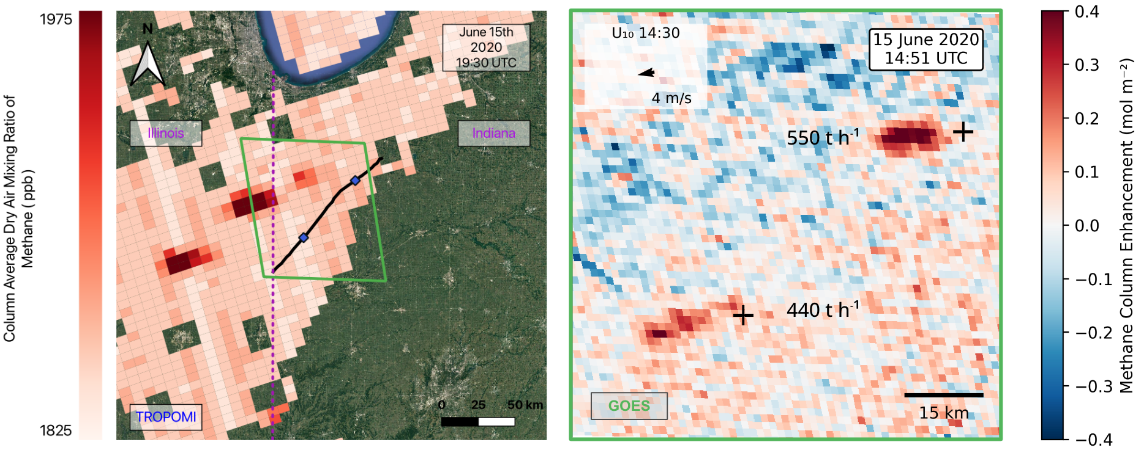

Figure S7. TROPOMI (left) and GOES (right) methane plume detections of simultaneous releases from a natural gas pipeline in Indiana (see Movie S3). The releases began at ~16:15 UTC on 15 June 2020 and lasted roughly one hour at rates of 440 t h^{-1} and 550 t h^{-1} (right panel inset). TROPOMI observed the two plumes 5 hours later, downwind over Illinois. The release points are block valve stations marked by blue diamonds (left) and black plus symbols (right). The pipeline path (black line, left) is from IndianaMap (last access: 6 October 2023). The GOES image domain is shown as a green inset box in the TROPOMI scene. Background imagery is from © (2023) Google Earth.

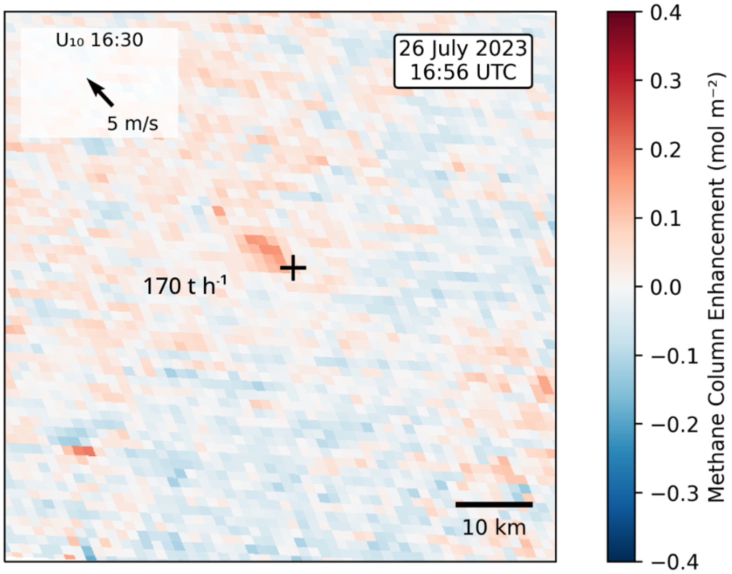

Figure S8. GOES detection of a large methane plume in the Permian basin. The plume was observed at (31.552°N, 103.843°W) on 26 July 2023 and identified independently with GOES, without prior information from other instruments.

Movie S1. 5-minute sequence of GOES (masked) methane plume retrievals for the 12 May 2019 EELL pipeline release. Background imagery is from © (2023) Google Earth.

Movie S2. 5-minute sequence of GOES (unmasked) methane plume retrievals for the 12 May 2019 EELL pipeline release.

Movie S3. 5-minute sequence of GOES methane plume retrievals for the 15 June 2020 simultaneous pipeline releases shown in Fig. S7.

Movie S4. 5-minute sequence of GOES methane plume retrievals for the 26 July 2023 Permian release shown in Fig. S8.

References for Supporting Information

IndianaMap (2023): https://www.indianamap.org/datasets/INMap::energy-pipelines-oil-and-gas/explore?location=40.748298%2C-86.648326%2C9.22, last access: 6 October 2023.
