# Supplementary figures and images for: Geostationary satellite observations of extreme and transient methane emissions from oil and gas infrastructure
Source: Proc Natl Acad Sci U S A. 2023 Dec 19;120(52):e2310797120. doi: 10.1073/pnas.2310797120 (PMC10756283; doi:10.1073/pnas.2310797120)

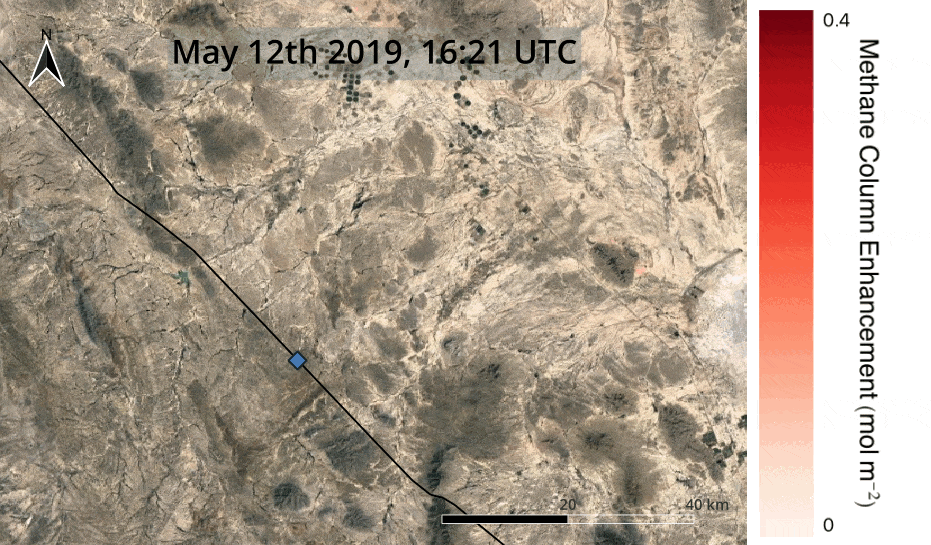

Supplement: Movie S1. — 5-minute sequence of GOES (masked) methane plume retrievals for the 12 May 2019 EELL pipeline release. Background imagery is from © (2023) Google Earth. [file pnas.2310797120.sm01.gif]

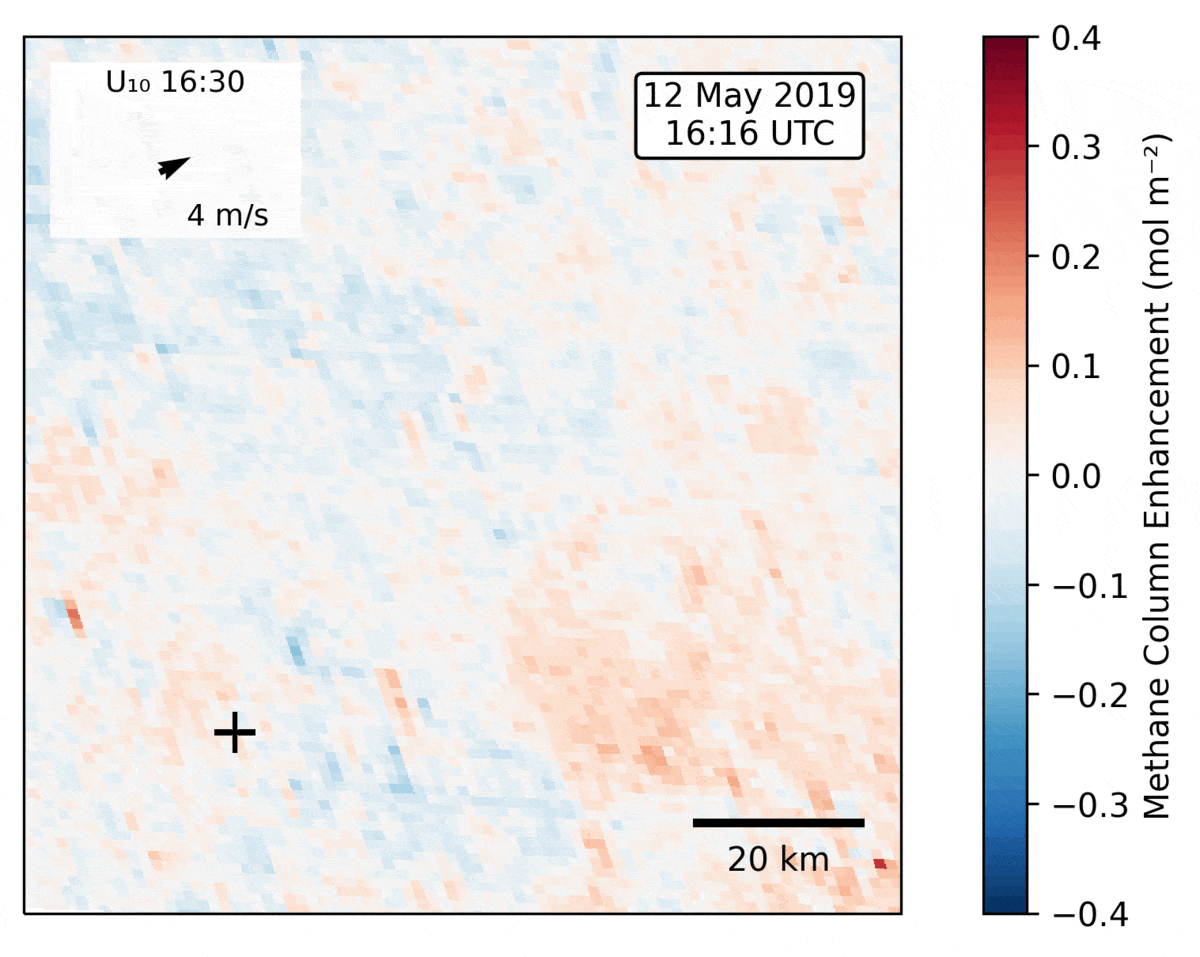

Supplement: Movie S2. — 5-minute sequence of GOES (unmasked) methane plume retrievals for the 12 May 2019 EELL pipeline release. [file pnas.2310797120.sm02.gif]

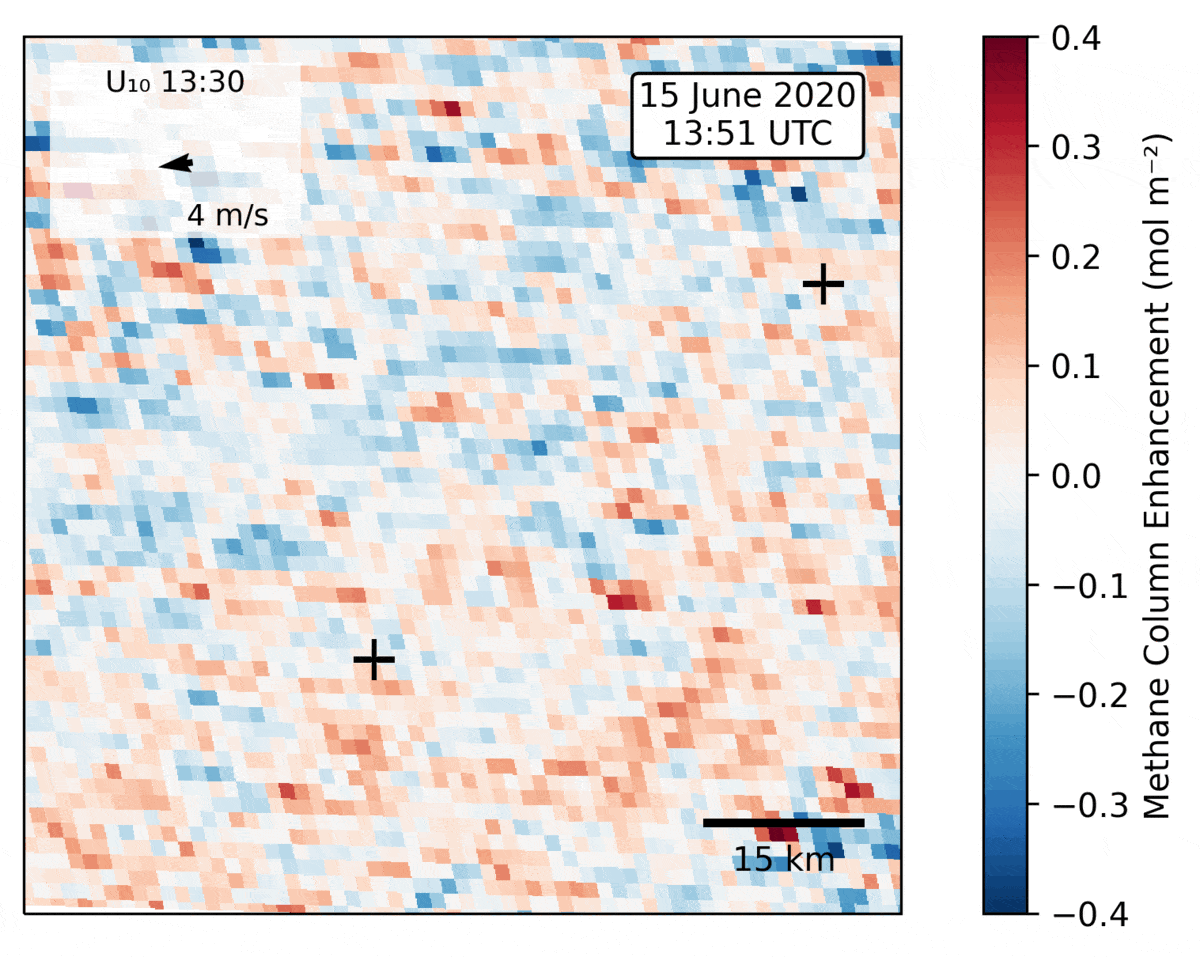

Supplement: Movie S3. — 5-minute sequence of GOES methane plume retrievals for the 15 June 2020 simultaneous pipeline releases shown in Fig. S7. [file pnas.2310797120.sm03.gif]

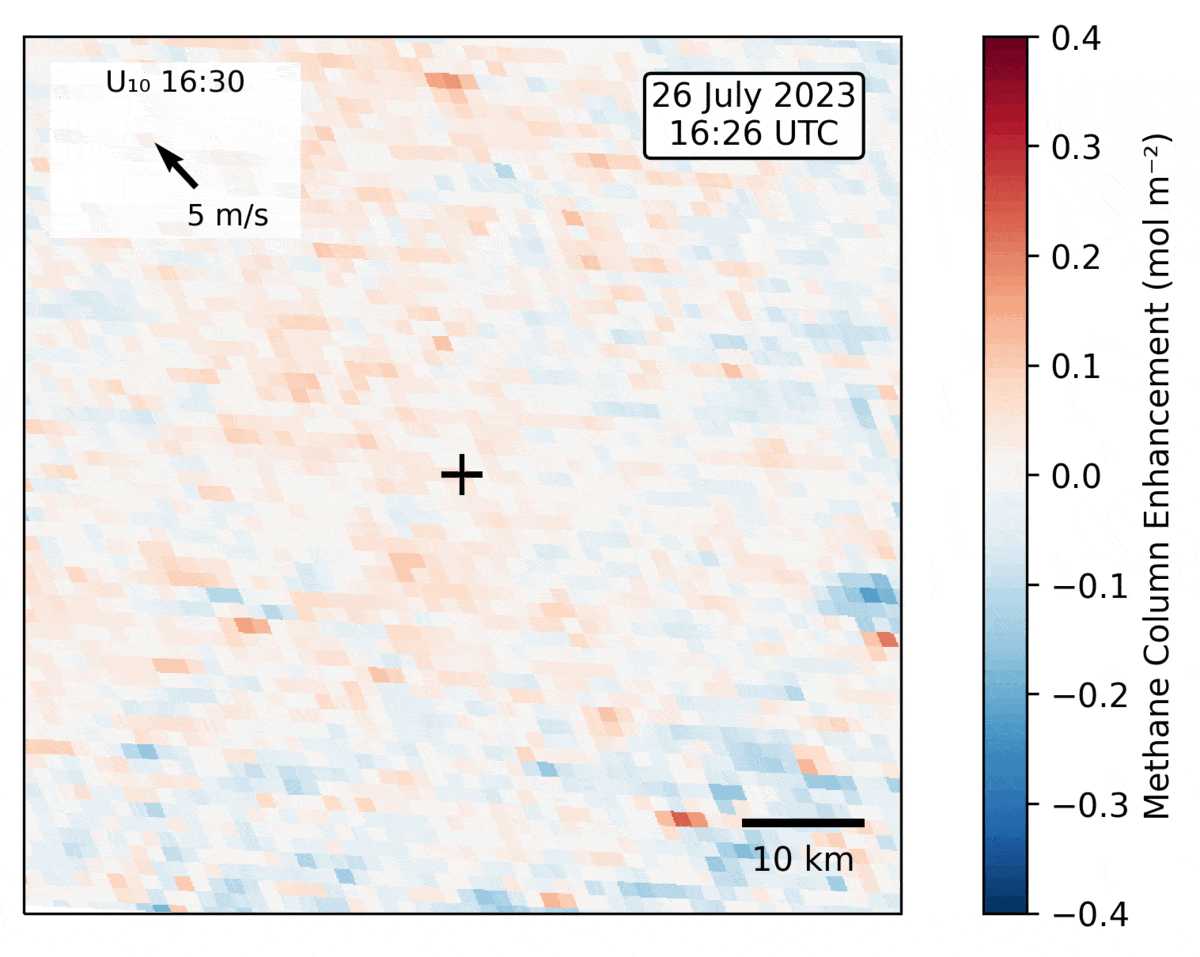

Supplement: Movie S4. — 5-minute sequence of GOES methane plume retrievals for the 26 July 2023 Permian release shown in Fig. S8. [file pnas.2310797120.sm04.gif]
